# A pilot randomized study of a telephone-based cognitive-behavioral stress-management intervention to reduce distress in phase 1 oncology trial caregivers

**DOI:** 10.1017/S1478951523000196

**Published:** 2023-10

**Authors:** Alaina L. Carr, Emily Bilenduke, Esmeralda Adolf, Elizabeth R. Kessler, Joanna J. Arch, Krista W. Ranby, Kristin Kilbourn

**Affiliations:** 1 Department of Psychology, University of Colorado Denver, Denver, CO, USA;; 2 Lombardi Comprehensive Cancer Center, Cancer Prevention and Control, Washington, DC, USA;; 3 Division of Medical Oncology, Department of Medicine, University of Colorado, Aurora, CO, USA; 4 Department of Psychology and Neuroscience, University of Colorado Boulder, Boulder, CO, USA

**Keywords:** Cancer, Caregivers, Phase I trials, Pilot projects, Stress-management

## Abstract

**Objectives.:**

Caregivers of adult phase 1 oncology trial patients experience high levels of distress and face barriers to in-person supportive care. The Phase 1 Caregiver LifeLine (P1CaLL) pilot study assessed the feasibility, acceptability, and general impact of an individual telephone-based cognitive behavioral stress-management (CBSM) intervention for caregivers of phase I oncology trial patients.

**Methods.:**

The pilot study involved 4 weekly adapted CBSM sessions followed by participant randomization to 4 weekly cognitive behavioral therapy sessions or metta-meditation sessions. A mixed-methods design used quantitative data from 23 caregivers and qualitative data from 5 caregivers to examine the feasibility and acceptability outcomes. Feasibility was determined using recruitment, retention, and assessment completion rates. Acceptability was assessed with self-reported satisfaction with program content and participation barriers. Baseline to post-intervention changes in caregiver distress and other psychosocial outcomes were assessed for the 8-session intervention.

**Results.:**

The enrollment rate was 45.3%, which demonstrated limited feasibility based on an a priori criterion enrollment rate of 50%. Participants completed an average of 4.9 sessions, with 9/25 (36%) completing all sessions and an 84% assessment completion rate. Intervention acceptability was high, and participants found the sessions helpful in managing stress related to the phase 1 oncology trial patient experience. Participants showed reductions in worry and isolation and stress.

**Significance of results.:**

The P1CaLL study demonstrated adequate acceptability and limited feasibility and provided data on the general impact of the intervention on caregiver distress and other psychosocial outcomes. Caregivers of phase 1 oncology trial patients would benefit from supportive care services; a telephone-based intervention may have more utilization and thus make a larger impact.

## Introduction

Caregivers of phase 1 oncology trial patients are a distressed population (Kessler et al. 2014; Kogan et al. 2022; Rezash et al. 2019). Caregiver distress stems from the multiple stressors related to the phase 1 oncology trial experience, as these trials are first in human safety studies of a new pharmacological agent (Weber et al. 2015). The trial design incorporates numerous tests or procedures to gather maximal safety and efficacy data to inform future drug development (Weber et al. 2015). Patients who enroll in phase 1 oncology trials have generally exhausted standard treatment approaches. Furthermore, the median survival of phase 1 oncology trial patients is approximately 5–9 months, and participation in these trials often precludes enrollment in hospice care (Cassel et al. 2016; Ferrell et al. 2017; Rezash et al. 2019; Sun et al. 2014). Therefore, caregivers are critical members of patients’ care teams to meet trial requirements.

Caregivers have higher levels of stress related to phase 1 oncology trial participation, travel requirements, family-related burdens, and end-of-life concerns compared to patients (Kessler et al. 2014; Rezash et al. 2019). Additionally, caregivers of phase 1 oncology trial patients reported worse coping compared to dementia caregivers and higher levels of anxiety, perceived stress, depressive symptoms, and impaired emotional regulation than population norms (Kessler et al. 2014). Thus, caregivers of phase 1 oncology trial patients are at risk for negative outcomes (low social support, mood, increased morbidity, and mortality) due to the myriad of physical, emotional, and mental stressors associated with the trial and demands of caregiving (Bevans and Sternberg 2012; Northouse et al. 2012). However, research to date on this caregiver population is cross-sectional (Kessler et al. 2014; Rezash et al. 2019) or exploratory (Kogan et al. 2022), and there are no longitudinal evidenced-based interventions focused on addressing the specific needs of this population.

Consistent evidence indicates that cognitive behavioral stress-management interventions (CBSM) are effective in reducing stress and improving positive affect, quality of life, coping, and benefit finding among various patient populations (Antoni et al. 1991, 2001; Penedo et al. 2021, 2007; Walsh et al. 2022; Wang et al. 2018). CBSM helps increase participant awareness of sources of stress, identify and challenge distorted thoughts, utilize adaptive coping skills, and manage physical tension (Antoni 2003; Antoni et al. 2006; Wang et al. 2018). CBSM combines cognitive behavioral techniques and relaxation training to show improved psychological adaption among various medical populations (Antoni et al. 1991, 2006, 2001; Gudenkauf et al. 2015; Kilbourn et al. 2011; Penedo et al. 2021, 2007; Walsh et al. 2022; Wang et al. 2018).

CBSM interventions have been adapted for caregivers of bone marrow transplant patients and hospice patients (Kilbourn et al. 2011; Laudenslager et al. 2015; Simoneau et al. 2017). An adapted CBSM pilot intervention for hospice caregivers, the Caregiver LifeLine (CaLL) study, focused on coping, social support, self-care, logistical issues, and bereavement as key content areas based on a qualitative needs assessment study with 36 hospice caregivers (Kutner et al. 2009). The CaLL study examined the feasibility and acceptability of a 10-week individual telephone counseling intervention to improve coping skills and integrate a sense of meaning into caregiver activities (Kilbourn et al. 2011). The CaLL intervention reduced symptoms of depression and improved the emotional and social quality of life among caregivers of patients receiving hospice care. The CaLL intervention was feasible and acceptable; post-intervention interview data indicated that hospice caregivers were satisfied with the intervention and telephone modality.

Emerging research has shown that mindfulness-based meditation interventions reduce negative affect and increase positive affect in the general population (Luberto et al. 2020) and reduce avoidant coping, psychological distress, and family caregiver burden in pilot studies of caregivers of advanced-stage cancer patients (Hofmann et al. 2011; Johns et al. 2020). Mindfulness-based meditation interventions include loving-kindness meditation, also known as metta-meditation, which refers to an unconditionally loving and kindness meditation technique directed toward all beings. Metta-meditation begins with a focus on the self and then a gradual expansion to a loved one, a neutral person, a difficult person, and all beings (Hofmann et al. 2011; Telke et al. 2022). Mindfulness-based meditation may provide useful strategies for targeting distress, experiential avoidance, and coping with the medical realities faced by long-term caregivers such as caregivers of phase 1 oncology trial patients when combined with evidenced-based treatments (Hofmann et al. 2011; Johns et al. 2020; Telke et al. 2022).

Several studies found that telephone-delivered interventions effectively reduce depression, distress, and burden among caregiver populations (Eisdorfer et al. 2003; Wilz et al. 2017; Wilz and Soellner 2016). Cancer caregivers face many barriers to face-to-face psychosocial interventions including logistical problems, lack of free time, reluctance to leave the patient alone for extended periods, cost, and caregiver health problems (Kilbourn et al. 2011; Laudenslager et al. 2015; Wilz and Soellner 2016). Telephone-delivered interventions are viable solutions to these barriers and can provide much-needed support services to cancer caregivers.

The Phase 1 CaLL (P1CaLL) intervention was adapted from the CaLL study (Kilbourn et al. 2011) to fit the identified needs of caregivers of phase 1 oncology trial patients. The adaption of the P1CaLL intervention involved a series of focus groups conducted by coauthors (E.R.K. and K.K.) with stakeholders consisting of phase 1 oncology trial caregivers to better understand their unique needs and to elicit direct feedback on the intervention content and mode of telephone delivery. The adapted CBSM session topics were further selected based on the relationship to the transactional model of stress and coping (Folkman and Lazarus 1984) and stakeholder focus group endorsement. The use of the telephone as a mechanism of intervention delivery was informed by stakeholder feedback, CaLL intervention participant satisfaction with the telephone delivery of the CaLL intervention (Kilbourn et al. 2011), and the potential to offer greater access and convenience by eliminating geographic and technologic barriers. The P1CaLL intervention included 4 adapted CBSM sessions along with a traditional cognitive behavioral therapy (CBT) component or with a metta-meditation component designed to enhance adaptive coping with managing stress and uncertainty, increasing caregiver self-efficacy in applying coping skills, and promote a sense of meaning and purpose (Supplementary Appendix 1).

The goals of this pilot randomized study were to (1) assess the feasibility and acceptability of a psychosocial intervention delivered by telephone and (2) provide a preliminary assessment of the general impact of the intervention on caregiver distress and other psychosocial outcomes.

## Methods

### Participants

The eligibility criteria included the following: (1) self-identification as a primary caregiver for a phase 1 oncology trial patient, (2) completion of the Patient Health Questionnaire (PHQ-4) (Kroenke et al. 2009) screener with a total score of ≥3, (3) stated willingness to comply with all study procedures and availability for the duration of the study, (4) ≥18 years old, (5) consistent access to a telephone, (6) ability to read and understand English, (7) absence of cognitive or psychiatric conditions prohibiting participation (significant developmental or intellectual disability), and (8) not enrolled in another psychosocial intervention research study. Study participation did not restrict potential participant engagement in any mental health services.

### Procedures

Oncology providers identified caregivers from a phase 1 oncology trial clinic. Eligibility screening and written informed consent occurred during patients’ treatment visits or provider appointments. After consenting to the study, the study staff administered participants the PHQ-4 to establish final eligibility. Participants were eligible if their PHQ-4 total score was ≥3 across all 4 items, which indicated mild to severe distress (Kroenke et al. 2009). After baseline assessment completion, a master’s level clinician provided participants with 8 individual weekly intervention sessions. Participants received 4 CBSM sessions over the telephone and then were randomized to receive 4 weekly CBT sessions or 4 weekly metta-meditation sessions. Nine weeks after enrollment, participants completed the online post-intervention assessment of acceptability and secondary outcome measures. A subsample (*n* = 5) of participants who completed all 8 intervention sessions and the post-intervention assessment completed an exit interview conducted by study staff (E.B.).

### P1CaLL intervention

Caregivers received a P1CaLL participant workbook which consisted of visual aids, homework activities, and in-session exercises for the first 4 CBSM sessions. Telephone-based session scheduling occurred around participant work and caregiving schedules. Participants received instructions to use their workbooks during each 45- to 60-min telephone session and to complete homework activities (relaxation exercises, pleasurable activities, and coping strategies) between sessions to enhance self-efficacy in skills.

**Fig. 1.**
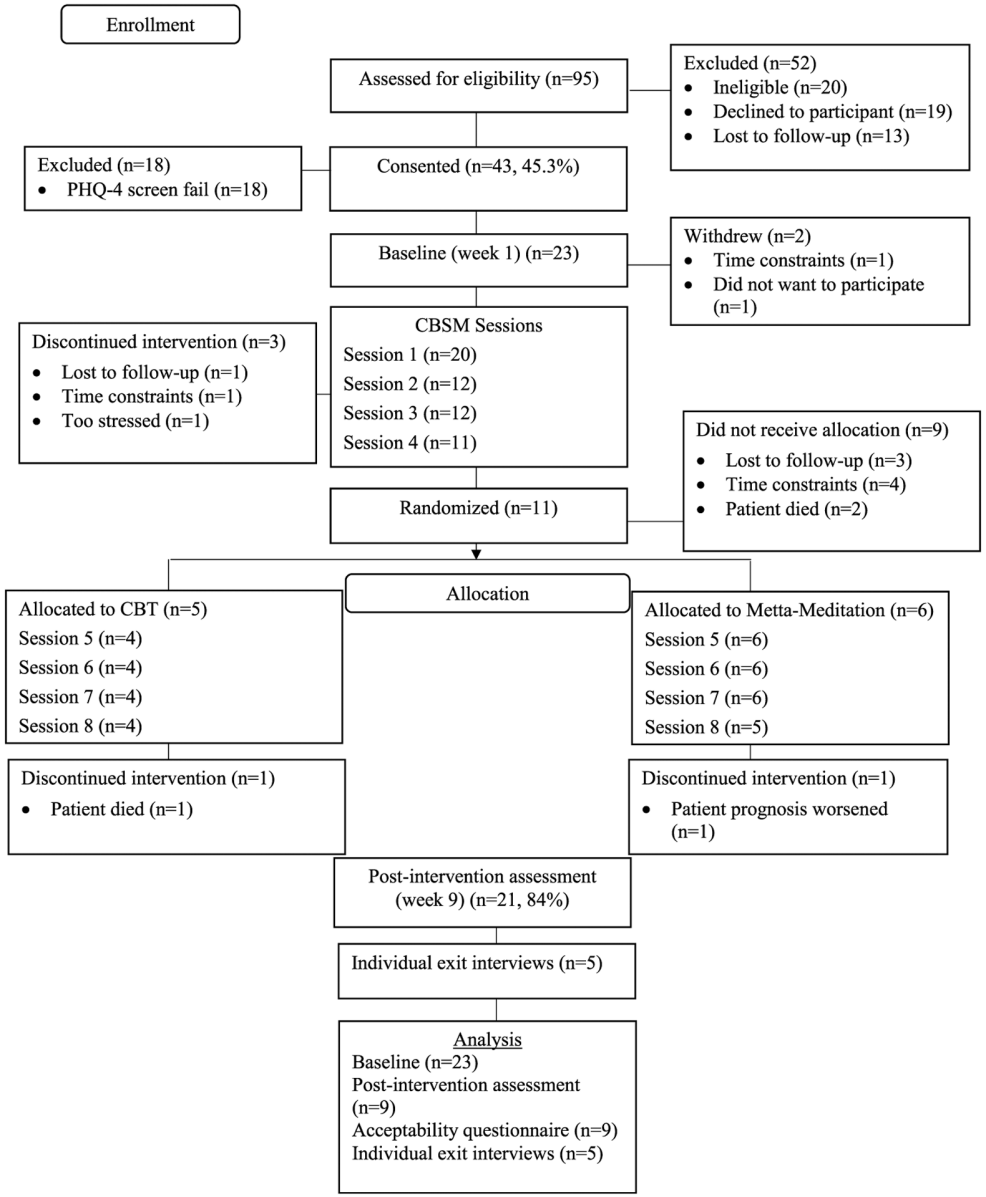
Consort study diagram.

After completing the first 4 CBSM sessions, participants were randomly allocated 1:1 to receive 4 CBT or 4 metta-meditation telephone-based sessions (Figure 1). Participants in the CBT intervention received emailed workbook exercises to use during sessions. Participants in the metta-mediation intervention received emailed meditation logs for between-session use and daily 15-min metta-meditation audio recordings. Caregivers who lost the person they cared for during the study were offered an optional grief and loss session. See Supplementary Appendix 1 for an overview of the P1CaLL sessions.

### Measures

Participants completed questionnaires at baseline and post-intervention via REDCap 9 weeks after enrollment. Sociodemographic questions included age, sex, race, marital status, employment status, relationship to the patient, and hours per week spent caregiving.

#### Primary outcomes

The primary outcomes were the feasibility and acceptability of the overall intervention. A priori benchmarks of feasibility were based on prior research of advanced cancer caregivers (Heynsbergh et al. 2018; Northouse et al. 2014) and defined by the recruitment of ≥50% of those approached enrolling in the study and by the retention of ≥50% of participants completing all sessions. Additional feasibility outcomes included assessment completion rates and the mean number of sessions delivered. Quantitative benchmarks of intervention acceptability included an 11-point Likert scale of acceptability at post-assessment with a goal of at least average ratings. The qualitative evaluation of the intervention acceptability addressed the timing of assessments, respondent burden, and subjective efficacy.

#### Secondary outcomes

Secondary outcomes were caregiver distress and other psychosocial variables measured at baseline and post-intervention. Changes in symptoms of distress were measured by the Depression Anxiety Stress Scale (DASS-21), a measure of 3 related negative emotional states of depression, anxiety, and stress (Lovibond and Lovibond 1995).

The Caregiver Reaction Assessment (CRA) included positive and negative dimensions of caregiver burden (Nijboer et al. 1999). The Positive Aspects of Caregiving (PAC) measured the psychosocial benefits of caregiving. The PAC included 2 subscale scores: self-affirmation and outlook on life, in addition to the overall PAC (Tarlow et al. 2004). The Marwit–Meuser Caregiver Grief Inventory (CGI) Short Form consisted of 18 items, and the total score and subscale scores measured factors of personal sacrifice burden, heartfelt sadness and longing, and worry and felt isolation (Marwit and Meuser 2005). The PHQ-4 measured symptoms of anxiety and depression (Kroenke et al. 2009). The Patient-Reported Outcomes Measurement Information System (PROMIS)-Ca Bank v1.0–Anxiety assessed symptoms of fear, worry, panic, tension, nervousness, and restlessness (Garcia et al. 2007). The 30-item Automatic Thought Questionnaire (ATQ) measured the frequency and belief of automatic negative thoughts associated with depression (Burgess and Haaga 1994). The 26-item Self-Compassion Scale (SCS) assessed thoughts, behaviors, and emotions associated with self-compassion (Neff 2003). The Dysfunctional Attitude Scale (DAS-24) measured the presence and intensity of dysfunctional attitudes and consists of 24 items; each item contained a statement and a 7-point Likert scale (1 = fully disagree, 7 = fully agree) (Power et al. 1994).

### Statistical analysis

Quantitative analyses conducted in the Statistical Package for the Social Sciences version 26 software included descriptive statistics and estimations of effect sizes due to the limited sample size (*N* = 25). Chi-squared tests identified systematic differences between those who enrolled and those who did not enroll in the trial. The Wilcoxon test (τ) for continuous variables and Fisher’s exact test for categorical variables were used to identify the impact of the intervention on secondary outcomes. Within-group effect sizes were calculated $\left(r=z/\sqrt{N}\right)$ (Fritz et al. 2012). Cohen’s guidelines for *r* effect sizes were utilized (*r* = .1 is small, *r* = .3 is medium, and *r* = .5 is large) (Cohen 1988; Fritz et al. 2012). The study was not powered to make comparisons between or across subjects. Participants were used as their own control, and baseline and post-intervention scores were compared to assess a general signal of the impact of the intervention on secondary outcomes.

The qualitative data were analyzed using applied thematic analysis, an inductive approach to identify broad themes and sub-themes within the exit interview data (Braun and Clarke 2006). The analysis involved systematically organizing data using open coding software ATLAS.ti v. 8 and continuous comparisons across coded data to extract salient themes.

## Results

### Feasibility

Forty-three (recruitment = 45.3%) of the 95 caregivers who approached consented to enroll in the study (Figure 1). Reasons for caregiver study refusal (*n* = 19) included self-reported adequate support networks (church, support groups, and family support), disinterest, or busy work and caregiver schedules. Eighteen caregivers were further excluded due to PHQ-4 total scores <3. Twenty-three of the 25 consented participants completed the baseline assessment. Twenty participants completed the first intervention session, the mean number of sessions delivered was 4.9 (SD = 4.2), and 9 participants completed all 8 intervention sessions (retention = 36%). On average, participants completed the 8 intervention sessions in 10.9 weeks (SD = 2.98). Twenty-one participants completed both assessment points (assessment completion rate = 84%). There were no significant differences in sociodemographic or clinical variables between participants who completed the intervention compared to those who did not (Supplementary Appendix 2). See Table 1 for the baseline sociodemographic and caregiver characteristics.

### Acceptability

**Fig. 2.**
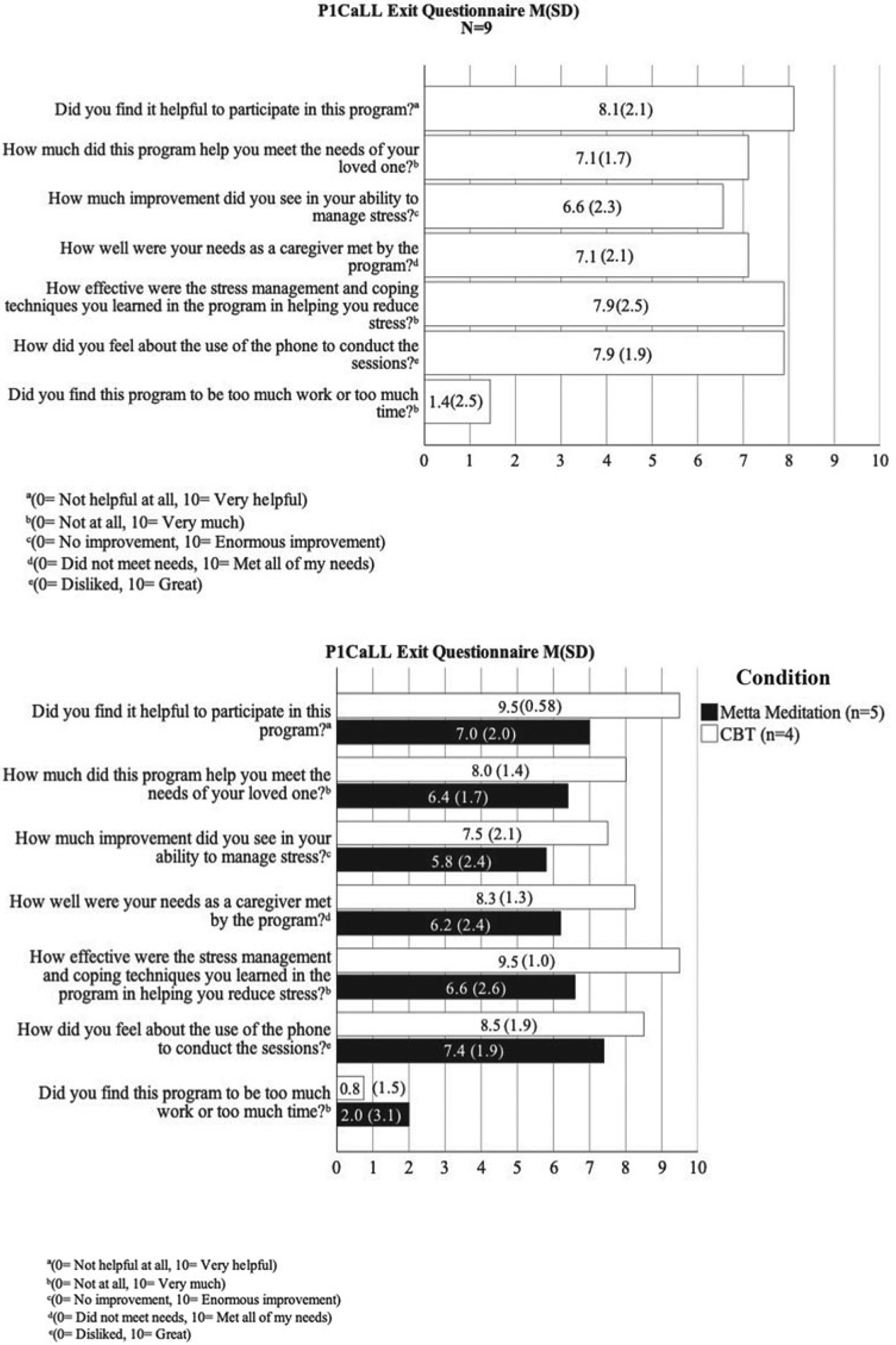
Program acceptability outcomes.

See Figure 2 for the average acceptability ratings among participants who completed all sessions and the post-assessment. A subset of these participants (*n* = 5) provided qualitative data regarding participant satisfaction with the session content and modality (Supplementary Appendix 3).

For many, the P1CaLL sessions increased awareness of adaptive stress management and coping relaxation exercises. Participants felt that session content normalized their caregiver experience, introduced beneficial stress-management skills, emphasized useful health behavior change strategies, and helped caregivers meet the needs of the person they care for. Several participants indicated that the stress-management skills learned in the program better prepared them for the stress affiliated with the phase 1 oncology trial. Several participants who completed the metta-meditation sessions explained that repeating phrases of love and kindness out loud helped decrease feelings of “hopelessness” and served as a daily reminder that they are “good people.” Many participants who completed the CBT intervention sessions reflected on how identifying unhelpful thinking styles was “powerful” in increasing their self-awareness of irrational thinking and the long-term unintended health consequences of this type of thinking. Many participants indicated the usefulness of the session content material depending on the relevance of the material to their caregiver roles. Participants provided neutral or negative descriptions of sessions that did not directly apply to their caregiving experience. For example, one caregiver said he did not relate to the social support session because he already had a strong social network. Overall, participants found the telephone-based modality of the P1CaLL program to be “convenient, time-saving, and practical.” All participants reported satisfaction with the total number and length of sessions.

Participants provided suggestions for improving the program. Several participants reported the telehealth and in-person community-based supportive resources provided at the end of the P1CaLL program did not target phase 1 oncology trial caregiver needs specifically, and caregivers’ demanding schedules conflicted with community program start times. These participants recommended including comparable community-based programs in future program iterations. Several participants reported a desire for initial face-to-face contact with the study interventionist before starting telephone-based sessions. Participants’ preference for initial face-to-face contact with the study interventionist was described as an “opportunity” to build rapport and improve participant “connection” with session material. One participant recommended scheduling sessions via text message. A full-time working caregiver expressed the need for a session that included strategies for managing guilt and distress associated with balancing work and caregiver-related responsibilities. Several participants recommended 1–2 booster sessions to review program information and relaxation exercises such as coping skills, metta-meditation practice, or CBT information.

### Secondary psychosocial outcomes

Baseline and post-intervention mean, SDs, and effect sizes are presented in Table 2. Data showed a large effect for CGI decrease in worry and felt isolation (*r* = −.52) and increase in DAS-24 self-control (*r* = .51). Medium effects were found for CRA health problems (*r* = .43) and a decrease in symptoms of DASS-21 stress (*r* = −.39). A small effect was found for a decrease in CRA financial problems (*r* = −.27), CRA lack of family support (*r* = −.20), CRA disrupted schedules (*r* = −.19), CGI heartfelt sadness and longing (*r* = −.12), SCS over identification (*r* = −.17), SCS common humanity (*r* = −.10), CGI total overall grief (*r* = −.28), and anxiety (PROMIS, *r* = −.21; DASS-21, *r* = −.16). Data yielded a small effect for increased SCS mindfulness (*r* = .22) and self-judgment (*r* = .20). No effects were found for symptoms of depression (DASS-21), anxiety (PHQ-4), positive benefit finding (PAC), personal sacrifice burden (CGI), degree of belief of automatic thoughts (ATQ), dysfunctional beliefs (DAS-24 achievement, DAS-23 dependency, and DAS-24 overall), overall compassion (SCS), SCS self-isolation, or SCS self-kindness.

## Discussion

The study was structured around 2 endpoints, designed to examine the feasibility, acceptability, and potential impact of the intervention on caregiver distress and other psychosocial outcomes. First, the P1CaLL pilot intervention program demonstrated limited feasibility and did not meet a priori recruitment or retention goals, with 45.3% of approached participants enrolling in the study and 36% (9/25) of participants completing all sessions. The mean number of sessions delivered was 4.9 (SD = 4.2), and the study yielded a high assessment completion rate (84%, 21/25).

Second, the P1CaLL program demonstrated adequate acceptability. Most caregivers were satisfied with the P1CaLL sessions and felt the CBSM sessions improved their ability to engage in adaptive coping during the phase 1 oncology trial experience. They noted increased confidence in applying program skills during stressful situations, such as patient appointments or experiencing high levels of distress. Interviewees emphasized the helpfulness of session topics depended on the individual relevance of specific content to their caregiving experience.

Although the study was not powered to assess efficacy or make comparisons between groups, the findings show promise for the P1CaLL intervention to reduce stress, worry, and felt isolation and improve caregiver health and feelings of self-control. Qualitative reports indicated that the stress-management sessions were adaptive to their needs. These findings are pertinent to distressed caregivers of phase 1 oncology trial patients by disrupting the cycle of maladaptive stress reactions from the phase 1 oncology trial experience and enhancing their ability to manage caregiver activities.

Future work should allow caregivers to choose session topics most applicable to their caregiving experience rather than standardizing topic order. Additionally, future work should consider incorporating content on balancing employment, caregiving demands, managing guilt, and the addition of booster sessions. Future iterations should consider adding a face-to-face meeting between counselors and participants before intervention initiation, offering text messaging as a convenient session scheduling feature, reassessing intervention duration, and establishing continuity when transitioning participants off the study intervention and into community-based settings.

Our lower-than-expected recruitment and retention rates highlight several challenges in engaging this unique caregiver population. We recruited caregivers of phase 1 oncology trial patients across the phase 1 oncology trial experience, which may have contributed to the number of consented participants that screen failed (41.9%, 18/43) as this may not have captured overall caregiver distress incidence and severity reflected in past research during the time of enrollment on phase 1 oncology trials (Kessler et al. 2014; Rezash et al. 2019). This was an unexpected finding, given that research examining cancer caregivers and research with phase 1 oncology trial caregivers has demonstrated that this population is highly distressed (Kessler et al. 2014; Rezash et al. 2019).

Attrition during the intervention was high, with busy caregiver schedules, caregivers feeling too stressed, and patient morbidity and mortality as the primary reasons for the noncompletion of sessions. This finding is consistent with other in-person and technology-based intervention studies for cancer caregivers that found similar intervention attrition rates ranging from 14% to 77% and reasons for attrition (length of intervention, worsening health status or death of patients, and caregiver schedules) (Fu et al. 2017; Heynsbergh et al. 2018; Kilbourn et al. 2011). Prior literature has shown that technology-based intervention duration between 6 and 8 weeks and content focused on the need of carers had lower attrition rates of 14–15% (Heynsbergh et al. 2018). However, the studies with lower attrition rates did not focus on caregivers of advanced cancer patients. It may be true for caregivers of phase 1 oncology trial patients that it is challenging to engage in longer interventions given the time-intensive nature of phase 1 trial participation and worsening patient prognosis. More research is needed to assess the optimal length of technology-based interventions combined with caregivers’ capacity to engage with interventions while managing the demands of trial participation.

The technological component of the P1CaLL intervention was challenging for a few patients. For example, some metta-meditation participants (*n* = 2) described difficulties downloading the REDCap audio recordings onto their smartphones, tablets, and computers. Reasons for technological difficulties included audio interface playback issues across various software applications.

A major challenge in this work was how to consider caregivers who lost the person they cared for during the intervention. More than 1/3 of the caregiver participants lost their patient to death before the post-assessment timepoint (patient died during the intervention: *n* = 4; patient died before post-intervention assessment: *n* = 2). Since the study did not specify how long patients needed to be enrolled in a phase 1 oncology trial, there was a great deal of variability in morbidity and mortality in the study group. Future work should consider standardizing patients’ duration in phase 1 oncology trials with a preference to enroll caregivers at the beginning of the trial for a more in-depth evaluation of the caregiver experience with the transition to and implications of the phase 1 oncology trial (Kessler et al. 2014).

Finally, the optional grief and loss session was only available to caregivers who lost their patients during the intervention phase of the study, and the caregivers who met these criteria (*n* = 4) declined the session. Grief and bereavement issues are a major part of the phase 1 caregiver experience since phase 1 clinical trials are often the last hope for patients who have exhausted standard treatment options. Future studies should consider how to incorporate support for caregivers who lose the people they provide care to (Caserta et al. 2019; Kilbourn et al. 2011).

### Study limitations

The generalizability of these results is subject to certain limitations. Most participants identified as white, female, and spousal caregivers, which although that is representative of the phase I clinic site (Kessler et al. 2014), the lack of heterogeneity in the sample offers less insight into how caregivers from other socioeconomic, racial, and ethnic groups cope with the demands of the trial experience. Future studies would benefit from recruitment efforts targeting male, non-spousal, and multiracial and ethnic caregivers. The study’s original intent aimed to compare the 2 arms on feasibility, acceptability, and preliminary efficacy potential. However, due to the small sample size, we were unable to make this comparison. This study yielded a small qualitative sample of caregivers that completed all program sessions, such that the qualitative analysis did not meet data saturation. Additionally, the qualitative interviews did not capture the experiences of caregivers who completed only a portion of the sessions, which would provide study staff with more insight into a broader range of caregiver perceptions of the intervention program. The small sample size, which allowed for testing our primary outcomes of feasibility and acceptability, did not allow tests of changes on secondary outcomes.

### Conclusion

A telephone-based CBSM intervention adapted to fit the specific needs of caregivers of phase 1 oncology trial patients was acceptable to most caregivers and demonstrated limited feasibility. Implementing this pilot study in this unique caregiver population was challenging due to caregivers’ multiple demands associated with caring for patients. However, most caregivers found that once enrolled, the program met their caregiving needs. Taken together, these preliminary findings suggest a role for targeted stress-management interventions in promoting coping and stress-management skills to assist caregivers in managing the high demands associated with phase 1 oncology trial patient participation.

## Supplementary Material

Appendix 3

Appendix 2

Appendix 1

## Figures and Tables

**Table 1.** Sociodemographic and caregiving characteristics (*N* = 23)

	Overall, *n* (%)
Age (years), *M* (SD)	56.7 (11.5)
Gender	
Female	17 (73.9)
Race	
White	20 (86.9)
Asian	1 (4.3)
American Indian or Alaskan Native	1 (4.3)
Black or African American	-
Other - Iberian	1 (4.3)
Hispanic	2 (8.6)
Patient cancer diagnosis	
Gastrointestinal	12 (52.2)
Genitourinary	1 (4.3)
Cutaneous	1 (4.3)
Lung	4 (17.4)
Head and neck	3 (13.0)
Sarcoma	1 (4.3)
Gynecological	1 (4.3)
Married/partnered	21 (91.3)
Dependent children	4 (17.4)
Other dependents	3 (13.0)
Family income^a^	
≤$50k	5 (21.7)
>$50k–≤ 100k	7 (30.4)
>100k	9 (39.1)
Education level	
High school diploma	2 (8.7)
Some college/associates degree	7 (30.4)
College/advanced degree	14 (60.9)
Employment status	
Employed full time	12 (52.2)
Employed part time	2 (8.7)
Retired	7 (30.4)
Unemployed	2 (8.7)
Relationship to patient	
Spouse	20 (87.0)
Child	2 (8.7)
Friend	1 (4.3)
Lives with patient	20 (87.0)
Duration of caregiving experience (years), *M* (SD)^b^	3.21 (3.6)
Patient died during study	6 (17.6)

a Two caregivers preferred not to answer.

b One caregiver responded, “less than a year.”

**Table 2.** Psychosocial outcomes (*N* = 9)

	*M* (SD)		Effect size (*r*)
Variable (*α*, reliability)	Baseline	Post-intervention	Baseline to post-intervention
DASS-21			
Anxiety (*α* = .56)	3.8 (4.2)	3.8 (4.8)	−.16
Depression (*α* = .92)	12.0 (11.0)	11.6 (11.3)	−.08
Stress (*α* = .88)	14.7 (7.9)	11.6 (8.0)	−.39
CRA (*α* = .73)			
Disrupted schedule	3.6 (0.9)	3.4 (0.7)	−.19
Financial problems	1.7 (0.7)	1.4 (0.6)	−.27
Lack of family support	2.1 (1.0)	1.9 (0.7)	−.20
Health problems	1.9 (0.6)	2.3 (0.7)	.43
Self-esteem	4.2 (0.5)	4.0 (0.3)	−.29
PAC (*α* = .71)			
Self-affirmation	20.8 (3.8)	20.7 (3.3)	−.01
Outlook on life	10.5 (2.5)	10.6 (3.4)	.08
Total	31.4 (4.9)	31.4 (4.9)	.00
CGI (*α* = .94)			
Heartfelt sadness and longing	16.8 (7.1)	16.2 (7.2)	−.12
Worry and felt isolation	15.7 (4.9)	13.3 (4.4)	−.52
Personal sacrifice burden	14.4 (7.0)	14.3 (7.2)	.04
Total	47.1 (17.1)	43.8 (17.2)	−.28
PHQ-4 (*α* = .83)	5.1 (2.8)	5.1 (3.2)	−.02
PROMIS (*α* = .92)	58.7 (5.7)	57.3 (4.8)	−.21
ATQ (*α* = .97)			
Frequency	53.2 (18.3)	56.6 (22.0)	.13
Degree of belief	53.3 (23.3)	64.7 (36.5)	.05
SCS (*α* = .94)			
Self-kindness	3.0 (1.1)	2.9 (0.9)	−.09
Self-judgment	3.3 (1.0)	3.4 (1.0)	.20
Common humanity	3.3 (0.9)	3.2 (0.5)	−.10
Self-isolation	3.2 (1.2)	3.1 (0.6)	−.03
Mindfulness	3.0 (0.7)	3.2 (0.8)	.22
Over identified	3.6 (0.9)	3.4 (0.9)	−.17
Total	3.2 (0.8)	3.2 (0.6)	.01
DAS-24 (*α* = .92)			
Achievement	2.7 (1.4)	2.6 (0.9)	−.08
Dependency	2.9 (1.1)	2.9 (1.1)	−.02
Self-control	2.9 (0.7)	3.5 (0.6)	.51
Total	69.7 (23.3)	73.4 (16.6)	.04

*r* = .1, small effect; *r* = .3, medium effect; *r* = .5, large effect (Fritz et al. 2012).

Abbreviations: ATQ, Automatic Thought Questionnaire; CGI, Caregiver Grief Inventory; CRA, Caregiver Reaction Assessment; SCS, Self-Compassion Scale; DAS, Dysfunctional Attitudes Scale; DASS, Depression Anxiety and Stress Scale; PAC, Positive Aspects of Caregiving; PHQ-4, Patient Health Questionnaire; PROMIS, Patient-Reported Outcomes Measurement Information System.
