# Supplementary material for: A pilot randomized study of a telephone-based cognitive-behavioral stress-management intervention to reduce distress in phase 1 oncology trial caregivers
Source: Palliat Support Care. Author manuscript; Available in PMC 2024 Feb 16. (PMC10544682; doi:10.1017/S1478951523000196)
Supplement: Appendix 3 [file NIHMS1903187-supplement-Appendix_3.doc]

**Appendix 3.** Acceptability of the Intervention Exemplar Quotes

| **Themes (definition)** | **Example Quotes** |
| --- | --- |
| Satisfaction with program content | *Since my husband's been diagnosed, I have noticed panic, the physical panic attacks, and I had never experienced that before… I think that practice of the relaxation and the coping strategies was that reminder of-- I practice them outside so when I do have those true panic moments, I can get through them. -1025* |
|  | *One thing was recognizing that I'm not in control of this entire process and that's okay…I think that the coping strategies convinced me that it was okay for me to not always have things go my way as to how I was expecting things to work out for my wife. - 1024* |
| Satisfaction with program modality | *We're pretty rural people, so there's really not anybody that I can talk to on a regular basis. Having somebody that you can talk to that will listen is vitally important… considering where I live, which is quite a ways away from [the hospital]. I think it was the only possible way to do it. -1010* |
|  | *I think [the] phone's more convenient for somebody with a busy schedule... And I don't think it's realistic for me to get to [academic medical institution’s name] once a week. That's just too much. -1015* |
| Program improvements | *Going from an individual interaction over the phone weekly to the best option of here's your replacement… a group that's the second and fourth Thursday from 4:00 to 5:00 …wasn't really an apples-to-apples alternative. -1015* |
|  | *It just was more of forgetfulness. And I think part of it is if we had scheduled something through texting, I probably would have got on scheduling it. -1025* |
