# Supplementary material for: A pilot randomized study of a telephone-based cognitive-behavioral stress-management intervention to reduce distress in phase 1 oncology trial caregivers
Source: Palliat Support Care. Author manuscript; Available in PMC 2024 Feb 16. (PMC10544682; doi:10.1017/S1478951523000196)
Supplement: Appendix 2 [file NIHMS1903187-supplement-Appendix_2.doc]

**Appendix 2. Sociodemographic and Caregiving Characteristics by Completion Status**

|  | **Intervention Session Non-Completers**  **n=14** | **Intervention Session Completers**  **n=9** | ***p*** |
| --- | --- | --- | --- |
| Age (years), M (SD) | 55.3 (12.8) | 58.9 (9.4) | .66 |
| Gender |  |  |  |
| Female | 11 (78.6) | 6 (66.7) | .64 |
| Race |  |  |  |
| White | 13 (92.9) | 7 (77.8) | .21 |
| Asian | 1 (7.1) | - | .42 |
| American Indian or Alaskan Native | - | 1 (11.1) | .39 |
| Black or African American  Other- Iberian | -  - | -  1 (7.1) | .39 |
| Hispanic | 1 (7.1) | 1 (7.1) | .74 |
| Patient cancer diagnosis |  |  | .78 |
| Gastrointestinal | 8 (67.1) | 4 (44.4) |  |
| Genitourinary | - | 1 (11.1) |  |
| Cutaneous | - | 1 (11.1) |  |
| Lung | 2 (14.3) | 2 (22.2) |  |
| Head & neck | 3 (21.4) | - |  |
| Sarcoma | - | 1 (11.1) |  |
| Gynecological | 1 (7.1) | - |  |
| Married/ partnered | 13 (92.9) | 8 (88.9) | .84 |
| Dependent children | 2 (14.3) | 2 (22.2) | 1.0 |
| Other dependents | 2 (14.3) | 1 (11.1) | .66 |
| Family income ^a^ |  |  | .56 |
| < $50k | 5 (35.7) | - |  |
| >$50k-<100k | 4 (28.6) | 3 (33.3) |  |
| >100k | 4 (28.6) | 5 (55.6) |  |
| Education level |  |  | .84 |
| High school diploma  Some college/ Associates degree | 1 (7.1)  4 (28.6) | 1 (11.1)  3 (33.3) |  |
| College/ advanced degree | 9 (64.3) | 5 (55.6) |  |
| Employment Status |  |  | .71 |
| Employed full time | 8 (57.1) | 4 (44.4) |  |
| Employed part time | - | 2 (22.2) |  |
| Retired | 4 (28.6) | 3 (33.3) |  |
| Unemployed | 2 (14.3) | - |  |
| Relationship to patient |  |  | .78 |
| Spouse | 12 (85.7) | 8 (88.9) |  |
| Child | 1 (7.1) | 1 (11.1) |  |
| Friend | 1 (7.1) | - |  |
| Lives with patient | 12 (85.7) | 8 (88.9) | .82 |
| Duration of caregiving experience (years), M (SD) ^b^ | 4.1 (4.2) | 1.8 (1.5) | .17 |
| Patient died during study | 4 (29.0) | 2 (22.2) |  |

^a^ Two caregiver preferred not to answer.

^b^ One caregiver responded, “less than a year.”
