# Supplementary material for: A pilot randomized study of a telephone-based cognitive-behavioral stress-management intervention to reduce distress in phase 1 oncology trial caregivers
Source: Palliat Support Care. Author manuscript; Available in PMC 2024 Feb 16. (PMC10544682; doi:10.1017/S1478951523000196)
Supplement: Appendix 1 [file NIHMS1903187-supplement-Appendix_1.doc]

**Appendix 1.** P1CaLL Program Session Overview

Cognitive-Behavioral Stress Management (CBSM):

1. Mind-Body Connection (Diaphragmatic Breathing)
2. Coping Skills (Progressive Muscle Relaxation)
3. Communication (Deep Breathing and Progressive Muscle Relaxation)
4. Social Support (Autogenic Relaxation)

Cognitive Behavioral Therapy (CBT):

1. Intro to CBT-Tracking Automatic Thoughts
2. Identifying Distorted Thoughts
3. Challenging Distorted Thoughts
4. Core Beliefs/Relapse Prevention

Metta-Meditation:

1. Intro to Meditation via Mindfulness of the Breath and Body-Noticing Critical Self Talk
2. Intro to Brief Loving-Kindness Meditation and Self-Care
3. Continuing with Additional Metta-Based Exercises to Cultivate Compassion for Oneself and Others and Mitigating Self-Criticism
4. Review/Plan for Future
